# Role of Leaf Traits in Driving Genotypic Diversity‐Mediated Associational Effects in Silver Birch

**DOI:** 10.1002/ece3.71768

**Published:** 2025-07-08

**Authors:** Juri A. Felix, Philip C. Stevenson, Julia Koricheva

**Affiliations:** ^1^ Department of Biological Sciences Royal Holloway University of London Egham UK; ^2^ Royal Botanic Gardens Kew UK; ^3^ Natural Resources Institute University of Greenwich Chatham UK

**Keywords:** associational effects, canopy cover, genotype, genotypic diversity, leaf traits, phenolic compounds, plant–herbivore interactions, silver birch

## Abstract

Trees growing in more diverse stands generally experience less herbivory than those in less diverse ones, potentially due to neighbourhood‐mediated variations in traits which influence leaf palatability. While numerous studies have assessed leaf trait responses to species diversity, the influence of genotypic diversity on leaf traits, and subsequent effects on herbivory, remains poorly understood. We investigated two genotypes of silver birch ( *Betula pendula* ) growing in single‐, 2‐, 4‐ and 8‐genotype mixture plots in the Satakunta birch clone diversity experiment in SW Finland. Our aim was to determine whether genotypic diversity causes leaf trait variation and whether these changes are linked to herbivory. We found no effect of genotypic diversity on specific leaf area (SLA) or concentrations of phenolic compounds, but increased canopy cover around the focal trees was associated with lower concentrations of some phenolics. Genotypic diversity had a significant effect on herbivory, with one of the genotypes suffering more herbivory in 2‐genotype mixture plots than in single‐genotype plots. Genotype identity was the strongest predictor of both leaf traits and herbivory. Two birch genotypes differed in concentrations of most phenolic compounds, SLA and herbivory. Leaf trait–herbivory relationships were also genotype specific, with only one of the two genotypes exhibiting a negative correlation between herbivory and phenolics. Our study demonstrates that genotypic diversity is a poor predictor of leaf traits and herbivory in silver birch and indicates the importance of genotype selection as a consideration when establishing herbivory‐resilient forests.

## Graphical abstract

We investigated two genotypes of silver birch ( *Betula pendula* ) growing in monocultures and genotype mixture plots to determine whether genotypic diversity causes leaf trait variation, and whether these changes are linked to herbivory. Genotypic diversity had a significant effect on herbivory, with one of the genotypes suffering more herbivory in 2‐genotype mixtures than in monocultures; however, this was not driven by the variation of leaf traits. We also show that leaf trait–herbivory relationships are genotype specific, as only one of the two genotypes exhibited a negative correlation between herbivory and phenolics.

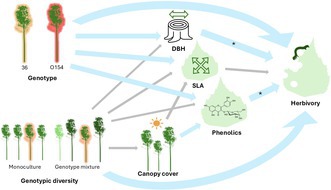

## 1 Introduction

Climate change and the spread of invasive species are intensifying the impacts of insect pests on trees, thereby threatening the ecosystem services, which forests provide (Jactel et al. 2019; Ramsfield et al. 2016; Seidl et al. 2018). Diversifying tree stands by increasing species or genotypic richness has been shown to influence the community structure of arthropods and plant–herbivore interactions and often reduces overall rates of arthropod herbivory (Barantal et al. 2019; Crawford and Rudgers 2013; Jactel et al. 2021; Koricheva and Hayes 2018; Stemmelen et al. 2022). The impacts of plant neighbourhood composition on herbivory are often described as associational effects, where associational resistance and associational susceptibility refer to neighbourhood‐mediated decreases or increases of herbivory, respectively (Barbosa et al. 2009). While species diversity‐mediated associational effects and their underlying mechanisms have been extensively studied in recent years (Jactel et al. 2021), few studies have examined how genotypic diversity might lead to associational resistance or associational susceptibility (Moreira et al. 2016).

One potential cause of associational effects may be variation of leaf traits, which alter leaf quality and rates of herbivory (Awmack and Leather 2002; Felix et al. 2023). In species mixtures, leaf trait variation may arise due to differences in environmental conditions, which respond to the specific species composition of a given neighbourhood (Davrinche and Haider 2021; Williams et al. 2020). For example, the intensity of light that reaches a focal tree's leaves could be reduced by the presence of neighbours that are taller or that have dense canopies, which can lead to increased specific leaf area (SLA) and reduced production of carbon‐based chemical defences, while the opposite may occur in the presence of shorter neighbours (Poeydebat et al. 2020; Williams et al. 2020). In addition, variation in environmental conditions can influence tree growth, which can alter investment into defences or the expression of certain leaf traits (Abdala‐Roberts et al. 2014; Herms and Mattson 1992).

A meta‐analysis by Koricheva and Hayes (2018) showed that the effects of plant genotypic diversity on arthropod abundance and diversity can be as strong as those mediated by plant species diversity. Despite this, the effects of tree genotypic diversity on leaf traits are poorly understood and have only been assessed in a handful of studies to date (Aubona et al. 2024; Moreira et al. 2014; Weih et al. 2021). It therefore remains unknown whether the genotypic diversity of neighbourhoods can cause the same degree of leaf trait variation as is commonly caused by species diversity.

We aim to address this knowledge gap using data collected from the Satakunta birch clone diversity experiment in southwest Finland, which contains eight genotypes of silver birch ( *Betula pendula* ) growing in single‐genotype or mixed‐genotype plots. Previous research using this experimental site found that birches growing in genotypically diverse plots experience associational resistance (Barantal et al. 2019) or associational susceptibility (Barton et al. 2015), depending on both the specific genotype examined and the type of herbivores. We build on this work by assessing whether leaf trait variation provides a mechanistic explanation for genotypic diversity‐mediated associational effects, specifically focussing on the effects of SLA and phenolic compounds.

Both SLA and phenolic compounds have previously been shown to vary in silver birch and other tree species depending on stand species richness (Castagneyrol, Moreira, and Jactel 2018; Felix et al. 2023; Muiruri et al. 2019; Poeydebat et al. 2020), which is often attributed to changes in light availability (Rozendaal et al. 2006). Here, we assess whether light availability also influences these traits in response to genotypic diversity by measuring variation in canopy cover (e.g., due to shorter or taller neighbouring genotypes) around the focal trees and investigating correlations with leaf trait measurements. We then assess whether correlations exist between leaf trait measurements and herbivory.

Plant–herbivore interactions in *Betula* spp. have been extensively studied, with differences in herbivory commonly linked to the leaf phenolics such as tannins and flavonoids, which are thought to act as chemical defenses (Himanen et al. 2010; Keinänen et al. 1999; Laitinen et al. 2000). Variation in phenolic profiles has also been found to be a contributing factor to stand species diversity‐mediated associational effects in silver birch (Castagneyrol, Jactel, and Moreira 2018; Poeydebat et al. 2020). However, some researchers have challenged this notion and argue that the primary role of leaf phenolics in *Betula* spp. and in other plant species is to protect leaves from UV‐radiation (Roberts and Paul 2006; Thitz et al. 2020). Our study contributes new insights into the ecological function of phenolics in silver birch by examining the correlations between foliar herbivory and leaf phenolic profile variation (be it due to genotype identity, genotypic diversity, or canopy cover).

Additionally, by comparing our results to those of Barton et al. (2015), the temporal trends in resistance of specific genotypes and the variation of genotypic diversity‐mediated associational effects over time were investigated. Finally, we assess how the above factors correlate with diameter at breast height (DBH) to determine whether genotypic diversity also influences tree growth.

To summarise, we use the Satakunta birch clone diversity experiment to explore the following questions:

1. What is the influence of genotype, genotypic diversity and their interaction on birch tree DBH, SLA, phenolics and herbivory?
2. Does plot‐level diversity influence canopy cover?
3. Does increasing canopy cover around the focal tree alter its leaf traits?
4. To what extent do DBH and leaf traits predict levels of leaf herbivory?

## 2 Methods

### 2.1 Experimental Site

The Satakunta birch clone diversity experiment was established in 2000 in southwest Finland (61.81° N, 21.67° E) on a clear‐cut site that was formerly planted with Norway spruce, and which is surrounded by mature boreal forest (https://www.sataforestdiversity.org/design). The experiment was planted with eight genotypes of silver birch (*Betula pendula*) micropropagated at the University of Helsinki and originating from southern Finland. These genotypes have known differences in their growth and susceptibility to biotic damage (Barton et al. 2015; Jia et al. 1997; Viherä‐Aarnio and Velling 2001). The experiment consists of forty‐eight 20 × 20 m plots planted with a single genotype or a mixture of two, four or eight genotypes (Figure S1). Treatments were randomly assigned to plots to minimise the confounding environmental effects (e.g., as a result of soil topography), and the position of each genotype within plots in genotypic mixtures was also randomised. Each plot originally contained 100 trees spaced 2 m apart; however, tree density was halved following a thinning treatment in 2013. In 2014, the evaluation of microsatellite loci revealed that most of the trees labelled as genotype ‘K1659’ had been mixed up during the planting stage and mostly represented genotype ‘V5952’ (Barantal et al. 2019). This error meant that plots containing ‘K1659’ genotypes were not planted evenly with respect to the number of each genotype represented. As a result, one of the two 4‐clone mixture plots and the four 8‐clone mixture plots sampled contained uneven proportions of each genotype (Figure S1).

### 2.2 Sampling Plan

Genotypes ‘O154’ and ‘36’ were selected as the focal trees in this study due to the known differences in susceptibility to herbivory, with Genotype 36 previously found to be more susceptible to chewing herbivore damage (Barton et al. 2015; Koricheva et al. 2025). Additionally, plots containing these two genotypes were well replicated throughout the experimental site, allowing for two independent assessments of genotypic diversity effects on leaf traits and herbivory.

Trees from each of the two focal genotypes were sampled in corresponding genotypic monocultures and two‐, four‐ and eight‐genotype mixture plots, all of which were replicated at least twice (Figure S1). Five trees per genotype were sampled in each plot, with directly adjacent trees and trees at the edge of the plot avoided when possible. The exception to this was the eight‐genotype plots, where 3–5 trees of each genotype were sampled depending on the availability of each genotype. All sampling and measurements occurred in mid‐July 2022 and were assumed to capture cumulative herbivory that had occurred in the time between leaf emergence in spring through to mid‐summer.

#### 2.2.1 Tree Growth and Canopy Cover

Diameter at breast height (DBH) for each focal tree was determined by measuring stem circumference at breast height (1.3 m from the ground) and dividing by π. Following Muiruri and Koricheva (2017), percentage canopy cover around the focal trees was assessed by walking around the trunk of each focal tree at a distance of 2 m and using a GRS densitometer (GRS, USA) to observe the canopy at 10 evenly spaced positions; at each position an observation of either ‘sky’ or ‘canopy’ was made, after which the sum of ‘canopy’ observations around each focal tree was multiplied by 10 to give an overall % canopy cover.

#### 2.2.2 Herbivory Assessment

Leaf herbivory, primarily due to chewing insects, was measured following a protocol in Muiruri et al. (2019). Four branches per tree were cut down with a branch pruner from heights of 3–6 m from each tree. For each branch, 25 short‐shoot leaves (leaves that had emerged during the spring) were inspected and categorised using the following damage categories: no damage, 0.1%–5%, 6%–25%, 26%–50%, 51%–75% and > 75%. Percent herbivory was calculated for each branch by multiplying the number of leaves in each category by the midpoint value of that category, and percent herbivory per tree was obtained by averaging percent herbivory values of the four branches assessed.

#### 2.2.3 Leaf Sampling and SLA Calculations

Ten undamaged leaves per tree were sampled from the same four branches used to assess herbivory and were stored under ice until further processing. A subset of five leaves per tree was photographed against a flat surface while still fresh (with petioles removed), and the area of each leaf was calculated using the software ImageJ (Schneider et al. 2012). All leaves were then dried using silica gel. Once dry, the same five leaves, which had been photographed, were weighed to give dry mass values. Specific leaf area (SLA) values for each tree were then calculated by dividing the leaf area by the dry weight for each leaf and then calculating the average. The dried leaves were then added to fresh silica gel for storage until chemical analysis.

### 2.3 Phenolic Compound Analysis

The 10 dried leaves from each sampled tree were pooled together and ground into a fine powder. Powdered leaves were then added to a solution of 70% acetone at a concentration of 20 mg/mL. The 70% acetone solution contained the reference compound chrysin at a concentration of 0.01 mg/mL as an internal standard. After 24 h, samples were centrifuged, and the supernatants were transferred into vials that were stored at −20°C until further analysis.

Each resulting sample was analysed using an LC–MS system consisting of an UltiMate 3000 Standard (SD) HPLC system (Thermo Scientific, USA) and a 100‐Hz diode array detector (DAD) hyphenated to a Velos Pro ion trap mass spectrometer (Thermo Scientific, USA). A Luna C18 column (150 mm × 3 mm i.d., 3 μm, Phenomenex, USA) held at 30°C was used as the stationary phase in combination with the following mobile phase (flow rate 400 μL/min): linear gradient of 0:90:10 to 90:0:10 [acetonitrile: water: acetonitrile +1% formic acid] over 20 min, followed by 90% acetonitrile for 5 min and then a return to initial conditions over 2 min, finally held for 3 mins. Phenolic compounds were detected in negative ionisation mode and were identified and classified by comparing retention times, masses and fragmentation patterns to literature sources and databases (Nerg et al. 2008; Paaso et al. 2017; Raal et al. 2015; Thitz et al. 2020; Tullus et al. 2021). In total, 53 phenolic compounds were identified, including condensed tannins, flavonoids, hydroxycinnamates, as well as a small number of monoaryl‐ and diaryl compounds and benzoic acids (Table S1).

### 2.4 Statistical Analysis

All statistical analyses were performed in R version 4.2.2 (R Core Team 2021). Linear mixed models (LMMs) were created with the packages *lme4* and *lmerTest* (Bates et al. 2015; Kuznetsova et al. 2017) to examine four questions (Table 1). Phenolic data were log‐transformed and herbivory data were log(y + 1)‐transformed prior to analysis to satisfy assumptions of normality. Plot was included as a random factor in all LMMs to avoid pseudoreplication arising from multiple trees being sampled from the same plot (Harrison et al. 2018).

**TABLE 1:** Linear mixed model (LMM) equations used in this study. All LMM equations follow the same format: Response variable ~ fixed variable(s) + (random factor). * denotes interaction term being included into the model in addition to the main effects of fixed variables.

Question	LMM equation
(1) Does genotype, genotypic diversity and their interaction influence DBH, leaf traits and herbivory?	“trait” ~ Genotypic diversity*Genotype + (1 | Plot)
(2) Does genotypic diversity influence canopy cover?	Canopy cover ~ Genotypic diversity + (1 | Plot)
(3) Does increasing canopy cover alter leaf traits?	“trait” ~ Canopy cover + (1 | Plot)
(4) What impact do leaf traits and DBH have on herbivory?	Herbivory ~ DBH + total phenolics + SLA + (1 | Plot)

First, the effect of genotype, genotypic diversity and their interaction on herbivory, leaf traits and DBH was examined.

Second, a separate LMM was then used to assess the effect of genotypic diversity on canopy cover to ascertain whether the canopy cover effect was independent of genotypic diversity effects.

Third, LMMs were run with canopy cover as a fixed factor and leaf herbivory, SLA, leaf phenolic subgroups and DBH as separate response variables to test whether canopy cover around focal birch trees affects herbivory and leaf traits for each of the two birch genotypes.

Fourth, the influence of tree growth and leaf traits on herbivory was assessed for each genotype using LLMs with herbivory as a response variable and SLA, DBH and total phenolics as fixed factors.

The effects of plot genotypic diversity and birch genotype on leaf phenolic profiles were further investigated using permutational multivariate analysis of variance (PERMANOVA) and principal coordinate analysis (PCoA) with the R package *vegan* (Oksanen et al. 2015). This allowed for data for all identified phenolic compounds to be analysed in a single model.

## 3 Results

### 3.1 Influence of Genotype and Genotypic Diversity on Leaf Traits and Growth

**FIGURE 1:**
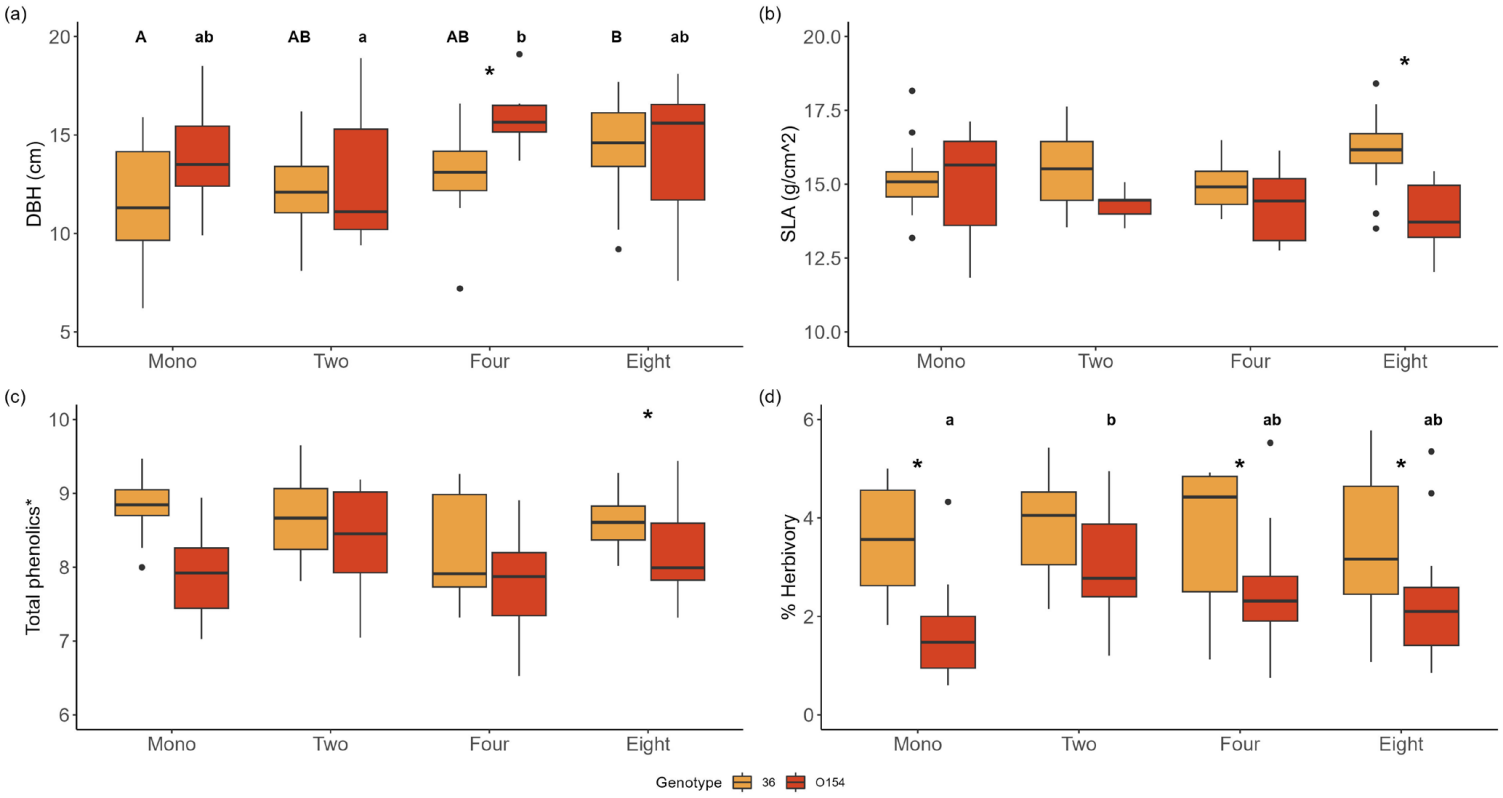
Influence of genotype and genotypic diversity on diameter at breast height (DBH), specific leaf area (SLA), total phenolics and % herbivory. *Total phenolics were measured using summed peak area in LC–MS relative to the internal standard of chrysin which was present in samples at a concentration of 0.01 mg/mL and was given a peak area value of 1. Results from post hoc tests are shown with different capital letters for significant differences between stand types, and with asterisks for significant differences between genotypes within the same stand type.

Birch genotypes O154 and 36 differed in DBH, SLA, herbivory, and phenolic profiles (Figure 1 and Table 2). Genotype O154 had greater average DBH and less herbivory than genotype 36. Total phenolics and all phenolic subgroups except condensed tannins were significantly influenced by genotype: foliar concentrations of total phenolics, quercetins, myricetins and kaempferols were on average higher in genotype 36, while concentrations of hydroxycinnamates were higher in genotype O154 (Table S2). Genotype O154 also had higher average SLA values than genotype 36, although this was only significant in 8‐genotype plots.

**TABLE 2:** Results of LMMs assessing (a) effects of silver birch genotype, genotypic diversity and their interaction on DBH, SLA, herbivory, total phenolics, and sub‐groups of phenolic compounds, and (b) the influence of genotypic diversity on canopy cover. Df = degrees of freedom (numerator, denominator). Significant results are shown in bold, marginally significant results are shown in bold italics. Significant *p* values are shown in bold with asterisks at the levels of “***” for *p* < 0.001, and “*” for *p* < 0.05, and marginally significant *p* values (*p* = 0.05–0.1) are shown in bold italics.

		Genotypic richness			Genotype			Genotypic richness × Genotype		
		Df	*F*‐value	*p*	Df	*F*‐value	*p*	Df	*F*‐value	*p*
(a)										
	DBH	3, 10.79	2.306	0.134	1, 16.36	5.215	0.036*	3, 16.54	1.581	0.232
	SLA	3, 11.81	0.192	0.900	1, 13.59	6.446	0.024*	3, 14.75	2.593	***0.092***
	% Herbivory	3, 97	3.035	0.033*	1, 97	33.610	< 0.001***	3, 97	1.838	0.145
	Total phenolics	3, 11.87	0.336	0.800	1, 12.79	7.394	0.018*	3, 13.68	0.440	0.728
	Flavonoids	3, 11.83	0.051	0.984	1, 13.13	59.969	< 0.001***	3, 14.19	0.275	0.842
	Quercetins	3, 11.88	0.139	0.935	1, 13.50	29.964	< 0.001***	3, 14.65	0.140	0.935
	Kaempferols	3, 11.55	0.446	0.725	1, 14.48	43.48	< 0.001***	3, 15.57	0.397	0.757
	Myricetins	3, 11.59	0.472	0.708	1, 14.34	163.610	< 0.001***	3, 15.46	0.552	0.538
	Condensed tannins	3, 11.73	0.963	0.442	1, 13.45	0.932	0.351	3, 14.61	1.809	0.190
	Hydroxycinnamates	3, 11.93	0.643	0.603	1, 13.49	7.627	0.016*	3, 14.65	0.350	0.790
(b)										
	Canopy cover	3, 14.87	0.774	0.527	—	—	—	—	—	—

Plot genotypic diversity had no effect on DBH or leaf traits, but significantly affected herbivory, particularly in genotype O154, which experienced almost twice as much herbivory in 2‐genotype mixtures as in single genotype plots (Table 2a and Figure 1d). No significant genotype*genotypic diversity interactions were found (Table 2a).

Mean canopy cover around the focal trees was not influenced by genotypic diversity (Table 2b) but was more variable in genotypically diverse plots than in single‐genotype plots (Figure S2).

### 3.2 Differences in Phenolic Profiles Between Genotypes

Results from PERMANOVA analysis indicated that genotype identity explained 62% of the variation in phenolic profiles among all birch trees sampled (Table 3). A significant interaction between genotype and genotypic diversity effects was also seen, which is likely due to the relatively high variation of phenolic profiles for genotype O154 in 4‐genotype plots.

**TABLE 3:** Results from PERMANOVA analysis assessing the influence of genotype, genotypic richness and their interaction on phenolic profiles. Significant factors shown in bold. Df = degrees of freedom, Pseudo‐F = measure of effect size analogous to F‐values generated by ANOVAs. Significant *p* values are shown in bold with asterisks at the levels of “***” for *p* < 0.001, and “**” for *p* < 0.01.

Factor	Df	Pseudo‐*F*	*R* ^2^	*p*
Genotype	1	183.87	0.62	< 0.001***
Genotypic richness	3	1.32	0.01	0.22
Genotype × Genotypic richness	3	3.25	0.03	0.003**

**FIGURE 2:**
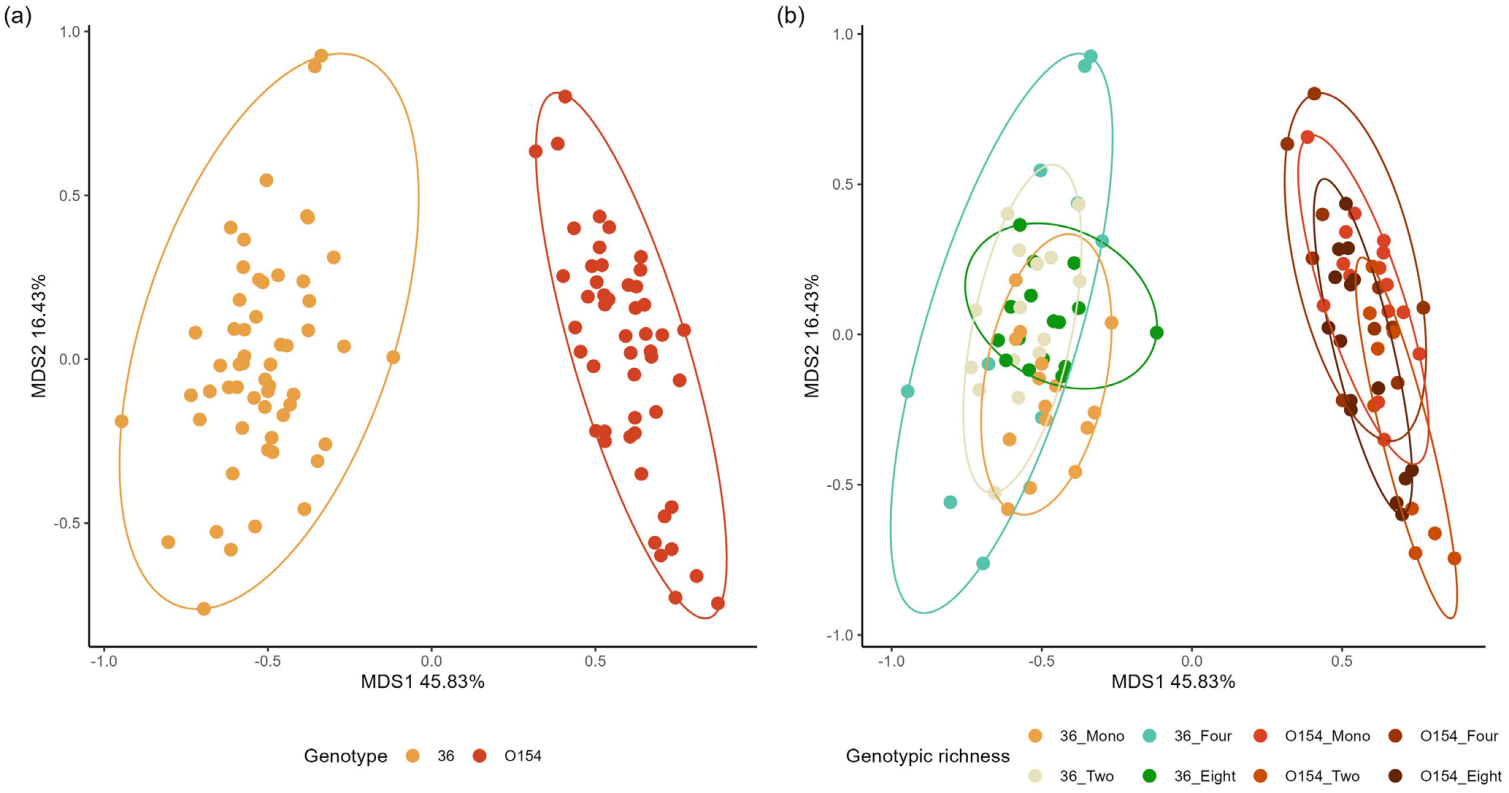
PCoA plots constructed using data on the relative concentrations of the 53 phenolic compounds identified in sampled birch leaves showing (a) the influence of genotype and (b) the influence of genotype and genotypic richness. Ellipses represent 95% confidence intervals of each group.

Principal Coordinate Analysis (PCoA) plots confirmed these findings by showing separation between genotypes along MDS1, accounting for 45.83% of total variation, but no separation between foliar phenolic profiles in trees growing in plots with different genotypic richness (Figure 2).

### 3.3 Effect of Canopy Cover on Leaf Traits

Higher canopy cover around focal trees was associated with higher SLA and decreased concentrations of total phenolics, flavonoids, quercetins and condensed tannins (Figure S3). However, canopy cover effects on leaf traits in genotype 36 were significant only for quercetins and marginally significant for total flavonoids, whereas in genotype O154, increased canopy cover around focal trees was associated with significantly reduced concentrations of total phenolics and flavonoids and a marginally significant reduction in condensed tannins (Table 4).

**TABLE 4:** Results of LMMs examining the influence of canopy cover on leaf traits. Df = degrees of freedom (numerator, denominator). Significant *p* values are shown in bold with asterisks at the levels of “*” for *p* < 0.05, and marginally significant *p* values (*p* = 0.05–0.1) are shown in bold italics.

Trait	Genotype					
	36			O154		
	Df	*F*‐value	*p*	Df	*F*‐value	*p*
SLA	1, 46.86	0.485	0.490	1, 43.12	0.154	0.697
Total phenolics	1, 45.58	1.032	0.315	1, 41.40	3.302	***0.076***
Flavonoids	1, 46.02	2.867	***0.097***	1, 44.50	1.058	0.309
Quercetins	1, 46.54	6.097	0.017*	1, 45.04	0.068	0.795
Kaempferols	1, 49.55	0.224	0.638	1, 46.49	1.283	0.263
Myricetins	1, 46.45	0.002	0.967	1, 47	0.360	0.551
Condensed tannins	1, 45.60	0.444	0.508	1, 43.94	3.692	***0.061***
Hydroxycinnamates	1, 48.07	1.532	0.222	1, 43.35	1.094	0.302

### 3.4 Effects of Leaf Traits and DBH on Herbivory

**FIGURE 3:**
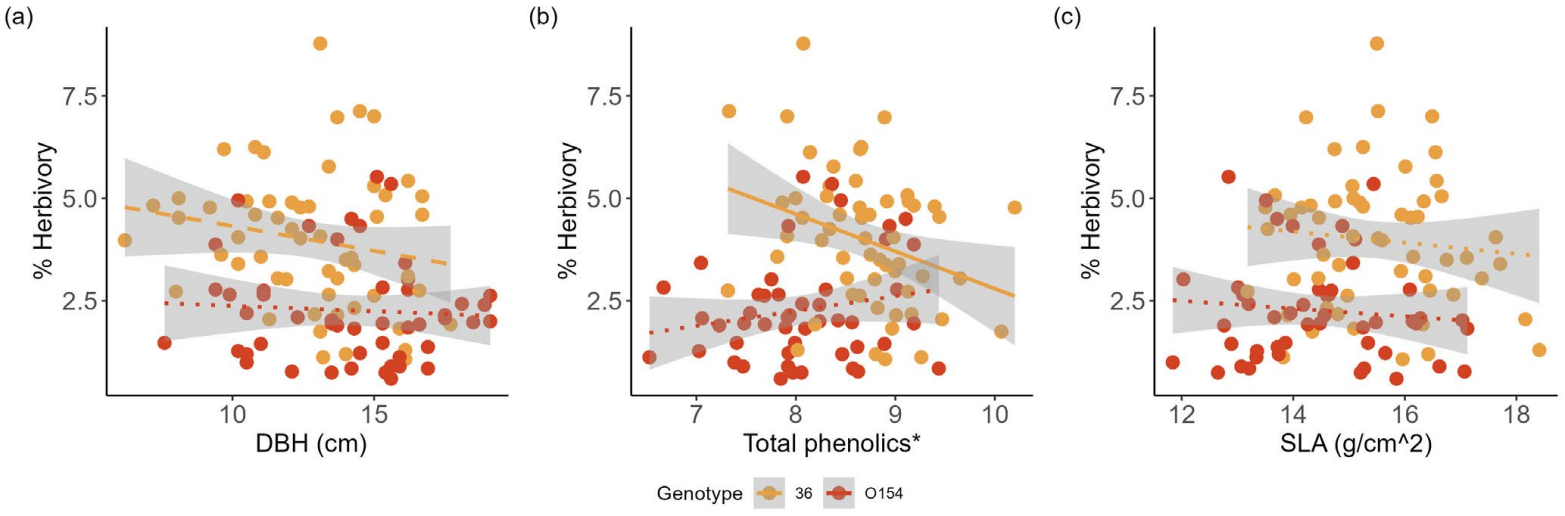
Relationship between % herbivory and (a) DBH, (b) total phenolics and (c) SLA. *Total phenolics were measured using summed peak area in LCMS in reference to chrysin which was present in samples at a concentration of 0.01 mg/mL and was given a peak area value of 1. Dotted lines indicate non‐significant relationships, dashed lines indicate marginally significant relationships, and solid lines show significant relationships.

Relationships between leaf traits, DBH and herbivory differed between the two genotypes (Figure 3). While no significant relationships were found in genotype O154, a significant negative relationship between total phenolics and herbivory and a marginally significant negative relationship between DBH and herbivory were found in genotype 36 (Table 5).

**TABLE 5:** Results of LMMs examining the influence of leaf traits on herbivory. Df = degrees of freedom (numerator, denominator). Significant *p* values are shown in bold with asterisks at the levels of ‘**’ for *p* < 0.01, and marginally significant *p* values (*p* = 0.05–0.1) are shown in bold italics.

Trait	Genotype					
	36			O154		
	Df	*F*‐value	*p*	Df	*F*‐value	*p*
DBH	1, 52	3.670	*0.061*	1, 44.40	0.194	0.662
Total phenolics	1, 52	7.304	0.009**	1, 32.31	1.299	0.263
SLA	1, 52	1.040	0.312	1, 39.33	0.184	0.670

## 4 Discussion

**FIGURE 4:**
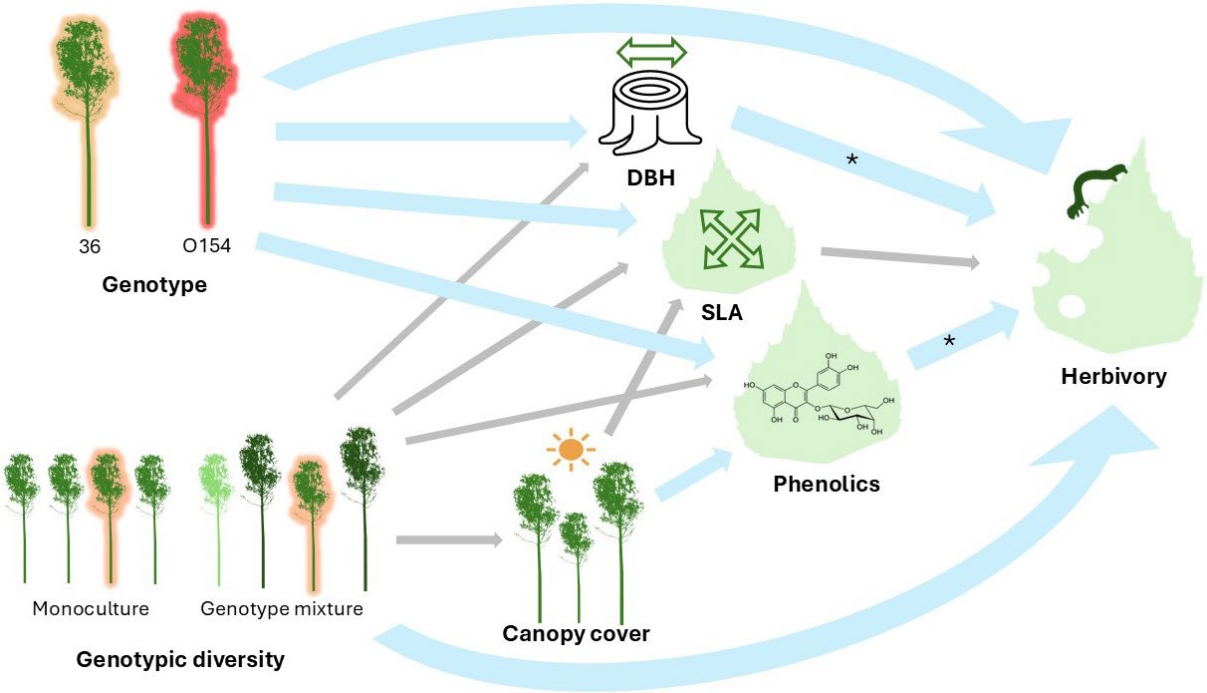
Conceptual diagram summarising the main findings of this study. Arrows show relationships between factors which were tested using LMMS: Thick blue arrows indicating that a significant relationship was found, thin grey arrows indicate that no significant relationship was found. Arrows with * indicate that significant relationships were found which were genotype specific (significant for genotype 36 but not genotype O154).

This study shows that leaf damage caused by insect herbivores on silver birches is predominantly determined by genotype, but can also be partially influenced by genotypic neighbourhood diversity. However, neither the effects of genotype identity nor that of neighbourhood genotypic diversity on herbivory were mediated by variation in tree growth, SLA or phenolic compounds (Figure 4).

Increased canopy cover around focal trees was weakly correlated with higher SLA and lower concentrations of phenolic compounds. However, variation in canopy cover was independent of plot‐level genotypic diversity, suggesting that genotypic diversity effects on leaf traits only occur on a finer spatial scale and may not be observed when assessed on a plot level. Moreover, the inconsistent relationship between phenolics and herbivory and the negative correlation between increasing canopy cover and phenolic compounds supports the theory that phenolic compounds in silver birch leaves primarily protect against UV radiation rather than herbivores (Thitz et al. 2020).

### 4.1 Differences Between Genotypes

The two birch genotypes examined in this study differed significantly in their growth (diameter at breast height) and phenolic profiles, in line with previous studies which have found high genotypic variation in the growth, physical leaf traits, and phenolic profiles of birches (Deepak et al. 2018; Keinänen et al. 1999; Laitinen et al. 2000; Paaso et al. 2017; Possen et al. 2014).

Genotype 36 was more susceptible to chewing insect damage than genotype O154, which is consistent with the findings of Barton et al. (2015) who analysed data gathered from the Satakunta birch clone diversity experiment 13 years prior to our study when the trees were still saplings. This comparison suggests that differences in susceptibility to herbivory between genotypes may be sustained across different ontogenetic stages.

### 4.2 Genotypic Diversity Effects on Herbivory

Herbivory varied with genotypic diversity for genotype O154, which experienced associational susceptibility when growing in 2‐genotype mixture plots. In contrast, herbivory on genotype 36 showed no response to plot‐level genotypic richness. Associational susceptibility of genotype O154 was not driven by variation in the measured leaf traits or differences in canopy cover, as neither was affected by genotypic diversity.

Barton et al. (2015) previously documented that both genotype O154 and genotype 36 experienced associational susceptibility to insect chewing damage in mixed‐genotype plots. This was attributed to spill‐over effects, that is, increases of herbivory on less susceptible species/genotypes when in close proximity to more susceptible species/genotypes (Berthelot et al. 2021; White and Whitham 2000). While our results only partially agree with this previous study, as we have shown that associational susceptibility can be lost over time (e.g., for genotype O154), the lack of any correlation between plot‐level leaf traits and herbivory supports the hypothesis that herbivore spill‐over from less resistant genotypes to more resistant genotypes could be responsible for associational susceptibility in genotype mixture plots.

### 4.3 Canopy Cover Effects

Increasing canopy cover around each focal tree was expected to shift leaf traits in directions associated with reduced light availability (Rozendaal et al. 2006; Williams et al. 2020). In this study, increased canopy cover was associated with higher SLA and lower concentrations of total phenolics, as well as several subclasses of phenolics. This pattern was only significant for total phenolics in the leaves of genotype O154 as well as quercetins for genotype 36, suggesting that the specific leaf compounds affected by canopy cover may differ with genotype identity.

Weak influence of canopy cover on leaf traits may indicate that measured % canopy cover was poorly correlated with light availability and other factors may have also been contributing to differences in the light level, such as genotype‐specific variation in canopy shape and self‐shading from upper branches in the sampled tree. Moreover, birches do not form dense canopies, which could result in relatively small differences in light intensity between high % canopy cover and low % canopy cover environments. Stronger effects of light and shading on birch SLA and leaf phenolics have been found in studies where light availability or UV‐radiation was measured directly (Lavola et al. 2013; Van Hees and Clerkx 2003).

Quercetin derivatives have higher photoprotective and antioxidant potential than myricetin and kaempferol derivatives (Lavola et al. 2013; Morales et al. 2010; Tegelberg et al. 2001; Thitz et al. 2020), so they were expected to show the strongest response to changes in canopy cover of the examined phenolic groups. This was the case, but only for genotype 36, which also contained higher concentrations of quercetins overall, which could indicate that genotype 36 is better adapted against UV‐induced stress than genotype O154.

Canopy cover is a function of neighbouring tree height and canopy shape in the immediate vicinity of each focal tree. As a result of the Satakunta birch clone diversity experiment's randomised planting scheme, taller/shorter clones are not evenly distributed across plots, which could explain why genotypic diversity on a plot level was not directly linked to canopy cover around the focal trees. Indeed, canopy cover was more variable in genotype mixture plots than it was in single genotype plots, likely due to a greater combination of taller/shorter neighbours around each focal tree in genotypically diverse plots (Figure S2). This may help explain why more predictable diversity effects on leaf traits have been observed at a highly localised scale (Davrinche and Haider 2021).

### 4.4 Tree Growth, Leaf Traits and Herbivory

Sustained defoliation reduces productivity and growth of trees (Visakorpi et al. 2021). This could explain why genotype 36, which experienced higher herbivory, had lower average DBH values than genotype O154, and why genotype 36 also exhibited a marginally significant correlation between lower DBH and higher rates of herbivory.

Interestingly, the sign of correlation between phenolic profiles and herbivory differed between genotypes, with a significant negative correlation in genotype 36 and a positive but non‐significant correlation in genotype O154. One possible explanation for this is that each genotype hosted distinct communities of herbivores that differed in their sensitivity to phenolic compounds (Barbehenn and Constabel 2011; Thitz et al. 2020). However, Barton et al. (2015) reported that most insect damage at the Satakunta birch clone experiment was from generalist chewers who tend to be sensitive to chemical defenses (Ali and Agrawal 2012). Moreover, since genotype 36 experienced more herbivory and had higher phenolics than genotype O154, there is little evidence to support this hypothesis. Nor is it likely that these opposing trends result from any differences in the induced production of flavonoids in response to defoliation between the genotypes, as genotype identity has been previously found to have little effect on the magnitude of induced defence responses in birches (Keinänen et al. 1999). In‐field observations of herbivore feeding patterns or feeding trails using collected leaf material may be required to better understand the link between leaf phenolics and herbivory in different birch genotypes.

The phenolic profiles of birch leaves are known to undergo seasonal shifts between spring and summer, with a reduction of soluble hydrolysable tannin concentration and an increase of insoluble, cell wall‐bound hydrolysable tannins in the time between budburst and midsummer (Salminen et al. 2001). Soluble hydrolysable tannins potentially play important roles in defence within birch leaves, but were not detected in the leaves analysed in our study, which had been sampled in mid‐July. It is therefore possible that this class of phenolic compound influenced herbivory in the spring and early summer months but had reduced in concentration to such a degree that they could not be detected in the leaves collected during our fieldwork in midsummer.

Alternatively, the results presented here could be interpreted as evidence for the growing consensus that leaf phenolics play only a partial role in mediating plant‐herbivore interactions alongside a suite of additional bottom‐up (e.g., physical and nutritional leaf traits) and top‐down (e.g., recruitment of natural enemies and parasitioids) factors, and thus may not always correlate directly with herbivore damage (Carmona et al. 2011; Thitz et al. 2020). Other leaf traits that were not measured in this study, such as triterpenoids, leaf thickness, trichome density, nitrogen or sugar content, in addition to phenological factors, the abundance and diversity of natural enemies, and genotype‐specific fungal communities may have all contributed to the different rates of herbivory for each genotype (Awmack and Leather 2002; Deepak et al. 2018; Färkkilä et al. 2023; Heimonen et al. 2017; Valkama et al. 2005).

### 4.5 Comparing the Effects of Genotypic Diversity and Species Diversity

In a comprehensive study of species diversity effects on birch leaf traits and herbivory across several tree diversity experiments, Poeydebat et al. (2020) found that birches growing in mixed‐species neighbourhoods expressed higher concentrations of phenolic compounds than those in monocultures, and that this was associated with a reduction in herbivory at northern latitudes. As fast‐growing pioneer trees, birches can quickly outgrow other species in newly established forest stands, and so can experience greater light availability in species mixtures than they do in monocultures. Increased light intensity promotes the production of phenolics due to higher rates of photosynthesis, or as an induced response to prevent UV‐damage, which in turn may reduce leaf quality and cause reductions of herbivory (Keski‐Saari et al. 2005; Lavola et al. 2013). Several specific flavonoids have been linked to anti‐feedant activities in silver birch leaves (Nerg et al. 2008; Thitz et al. 2020), and could be partially responsible for the trends described by Poeydebat et al. (2020).

No evidence of the above‐described mechanism occurring in genotypically diverse neighbourhoods was found. Despite finding that birches growing within less shaded environments (due to low canopy cover) had higher concentrations of phenolic compounds, % canopy cover was not mediated by genotypic richness and was not correlated with any detectable associational effects. This comparison complements the findings of Barantal et al. (2019), who described contrasting effects of tree species and genotypically diversity on birch leaf miners, and showed that only species diversity leads to reductions in leaf miner abundance.

Furthermore, our results show that relationships between phenolics and herbivory in birches may be genotype‐specific, and that higher concentrations of phenolics do not necessarily reduce herbivory. This finding implies that the negative relationships between phenolic concentrations and herbivory such as those described by Poeydebat et al. (2020) may not be causal, and could help explain why several other tree‐diversity studies have failed to find consistent relationships between species richness, leaf phenolics, and herbivory (Castagneyrol, Jactel, and Moreira 2018; Ferlian et al. 2020; Moreira et al. 2017).

## 5 Conclusion

In conclusion, our study finds that genotypic diversity can lead to associational susceptibility in silver birch, but that this is not mediated by variation in the measured leaf traits, as genotypic diversity had no effect on SLA or phenolic compounds. This may be partially explained by the effects of canopy cover, which exerted a weak but consistent effect on leaf traits, consistent with reduced light availability. Notably, this effect appears to be a function of immediate neighbours rather than plot‐level diversity.

Additionally, we found that leaf phenolic profiles can vary substantially between birch genotypes, yet their relationship with herbivory is inconsistent. This raises questions about the defensive function of phenolics in silver birch and highlights the limited understanding of the link between leaf traits and herbivory.

## Author Contributions

**Juri A. Felix:** conceptualization (lead), data curation (lead), formal analysis (lead), methodology (equal), writing – original draft (lead), writing – review and editing (equal). **Philip C. Stevenson:** methodology (supporting), supervision (equal), writing – review and editing (equal). **Julia Koricheva:** conceptualization (equal), data curation (equal), formal analysis (supporting), methodology (equal), supervision (equal), writing – review and editing (equal).

## Conflicts of Interest

The authors declare no conflicts of interest.

## Supporting information


Data S1.

## Data Availability

The data and code used in this publication is available on Zenodo https://zenodo.org/records/15083615.
