# Supplementary material for: Role of Leaf Traits in Driving Genotypic Diversity‐Mediated Associational Effects in Silver Birch
Source: Ecol Evol. 2025 Jul 8;15(7):e71768. doi: 10.1002/ece3.71768 (PMC12237825; doi:10.1002/ece3.71768)
Supplement: Supplementary file 1 — Data S1. [file ECE3-15-e71768-s001.docx]

**Role of leaf traits in driving genotypic diversity-mediated associational effects in silver birch – supporting information**

The following Supporting Information is available for this article:

**Figure S1:** Plot map with the layout of the Satakunta birch clone diversity experiment.

**Table S1:** List of phenolic compounds identified in birch leaves using LC-MS.

**Table S2**: Mean values of measured traits for silver birch genotypes ‘O154’ and ‘36’ growing in single genotype plots.

**Figure S2:** Canopy cover around focal trees of two birch genotypes growing in monocultures and, 2-, 4-, or 8-genotype plots.

**Figure S3**: Regression plots showing the influence of % canopy cover on birch leaf traits.

**
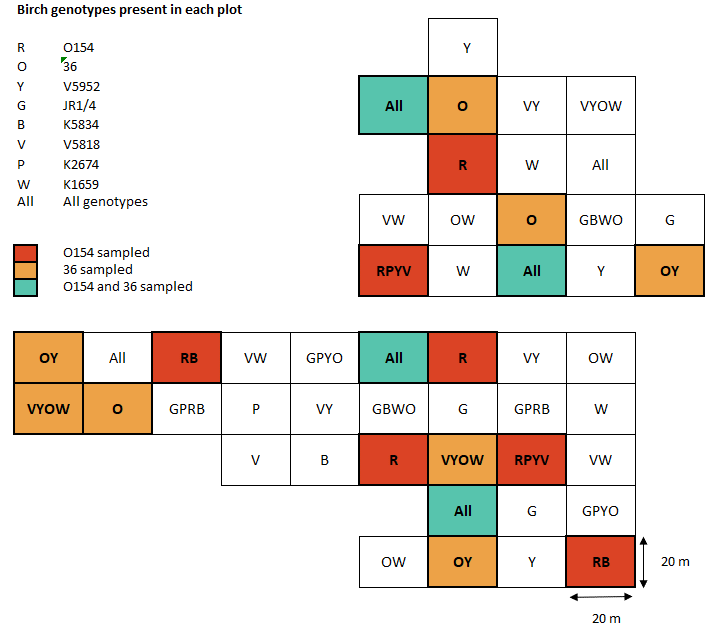
**

**Figure S1:** Plot map with the layout of the Satakunta birch clone diversity experiment showing which genotypes were present in each plot. Coloured areas indicate which plots were sampled in this study: Genotype 36 was sampled in orange plots, genotype O154 was sampled in red plots, and both genotypes were sampled in green plots.

| **Table S1:** List of phenolic compounds identified in birch leaves using LC-MS. *m/z* = mass/charge, RT = retention time (mins), MS2 = main fragmentation ions. | | | | | | |
| --- | --- | --- | --- | --- | --- | --- |
|  |  |  |  |  |  |  |
| **Assigned compound or isomer** | **Molecular formula** | **Ion** | ***m/z*** | **RT** | **MS2 fragments** | **class** |
| Prodelphinidin B4 | C_30_H_26_O_14_ | [M-H]^-^ | 609.13 | 2.25 | 591, 515, 483, 441, 423, 305 | Condensed tannin |
| Prodelphinidin B3 | C_30_H_26_O_13_ | [M-H]^-^ | 593.14 | 2.28 | 467, 441, 425, 407, 289 | Condensed tannin |
| Dihydrocoumaric acid glucoside | C_15_H_20_O_8_ | [M-H]^-^ | 327.20 | 2.39 | 179, 147 | Hydroxycinnamates |
| Caffeoylquinic acid | C_16_H_18_O_9_ | [M-H]^-^ | 353.10 | 2.40 | 191, 179 | Hydroxycinnamates |
| Dihydrocoumaric acid glucoside | C_15_H_20_O_8_ | [M-H]^-^ | 327.20 | 2.83 | 179, 147 | Hydroxycinnamates |
| Prodelphinidin B4 | C_30_H_26_O_14_ | [M-H]^-^ | 609.18 | 3.32 | 591, 515, 483, 441, 423, 305 | Condensed tannin |
| Prodelphinidin B3 | C_30_H_26_O_13_ | [M-H]^-^ | 593.16 | 3.36 | 467, 441, 425, 407, 289 | Condensed tannin |
| Gallic acid hexoside | C_13_H_16_O_10_ | [M-H]^-^ | 331.16 | 3.41 | 167 | phenolic acid |
| Protocatechuic acid hexoside | C_13_H_16_O_9_ | [M-H]^-^ | 315.10 | 3.82 | 225, 165, 153 | phenolic acid |
| (Epi)gallocatechin | C_15_H_14_O_7_ | [M-H]^-^ | 305.08 | 4.11 | 287, 261, 221, 219, 179 | Flavonoid |
| Prodelphinidin B3 | C_30_H_26_O_13_ | [M-H]^-^ | 593.18 | 4.36 | 467, 441, 425, 407, 289 | Condensed tannin |
| Caffeoylquinic acid | C_16_H_18_O_9_ | [M-H]^-^ | 353.14 | 4.56 | 191, 179 | Hydroxycinnamates |
| Prodelphinidin T3 | C_45_H_38_O_19_ | [M-H]^-^ | 881.19 | 4.72 | 863, 755, 711, 695, 593, 543, 467, 425, 407, 289 | Condensed tannin |
| Procyanidin B1-6 | C_30_H_26_O_12_ | [M-H]^-^ | 577.20 | 4.83 | 559, 451, 425, 407, 299, 289 | Condensed tannin |
| Dihydrocoumaric acid glucoside | C_15_H_20_O_8_ | [M-H]^-^ | 327.13 | 4.91 | 179, 147 | Hydroxycinnamates |
| Coumaroylquinic acid | C_16_H_18_O_8_ | [M-H]^-^ | 337.15 | 4.93 | 191, 173, 163 | Hydroxycinnamates |
| Caffeic acid hexoside | C_15_H_18_O_9_ | [M-H]^-^ | 341.09 | 5.21 | 323, 195, 163 | Hydroxycinnamates |
| Prodelphinidin B3 | C_30_H_26_O_13_ | [M-H]^-^ | 593.21 | 5.22 | 467, 441, 425, 407, 289 | Condensed tannin |
| Procyanidin B1-6 | C_30_H_26_O_12_ | [M-H]^-^ | 577.26 | 5.30 | 559, 451, 425, 407, 299, 289 | Condensed tannin |
| Coumaroylquinic acid | C_16_H_18_O_8_ | [M-H]^-^ | 337.18 | 5.41 | 191, 173, 163 | Hydroxycinnamates |
| Caffeoylquinic acid | C_16_H_18_O_9_ | [M-H]^-^ | 353.09 | 5.42 | 191, 179 | Hydroxycinnamates |
| Coumaric acid glucoside | C_15_H_18_O_8_ | [M-H]^-^ | 325.17 | 5.49 | 163, 119 | Hydroxycinnamates |
| Catechin | C_15_H_14_O_6_ | [M-H]^-^ | 289.15 | 5.63 | 245, 205, 179 | Flavonoid |
| Prodelphinidin B3 | C_30_H_26_O_13_ | [M-H]^-^ | 593.10 | 5.63 | 467, 441, 425, 407, 289 | Condensed tannin |
| Caffeic acid hexoside | C_15_H_18_O_9_ | [M-H]^-^ | 341.09 | 5.65 | 323, 195, 163 | Hydroxycinnamates |
| Coumaroylquinic acid | C_16_H_18_O_8_ | [M-H]^-^ | 337.18 | 6.35 | 191, 173, 163 | Hydroxycinnamates |
| Myricetin 3-O-galactoside/glucoside | C_21_H_20_O_13_ | [M-H]^-^ | 479.19 | 6.53 | 317, 316 | Flavonoid |
| Myricetin 3-glucuronide | C_21_H_18_O_14_ | [M-H]^-^ | 493.13 | 6.69 | 447, 317, 311 | Flavonoid |
| Quercetin galloylglucoside | C_28_H_24_O_16_ | [M-H]^-^ | 615.19 | 6.82 | 463, 301 | Flavonoid |
| Coumaroylquinic acid | C_16_H_18_O_8_ | [M-H]^-^ | 337.15 | 6.85 | 191, 173, 163 | Hydroxycinnamates |
| Myricetin-3-arabinofuranoside/pentoside | C_20_H_18_O_12_ | [M-H]^-^ | 449.11 | 7.12 | 317, 316 | Flavonoid |
| Myricetin 3-O-rhamnoside (Myricitrin) + Quercetin 3-O-galactoside (hyperoside) | C_21_H_20_O_12_ | [M-H]^-^ | 463.15 | 7.20 | 317, 316, 301, 300 | Flavonoid |
| Quercetin 3-O-glucuronide | C_21_H_18_O_13_ | [M-H]^-^ | 477.18 | 7.34 | 301 | Flavonoid |
| kaempferol 3-glucoside/galactoside | C_21_H_20_O_11_ | [M-H]^-^ | 447.17 | 7.60 | 419, 401, 327, 315, 285, 284, 255 | Flavonoid |
| Quercetin-3-O-pentoside | C_20_H_18_O_11_ | [M-H]^-^ | 433.19 | 7.79 | 301, 300 | Flavonoid |
| Phenethyl rutinoside | C_20_H_30_O_10_ | [M-H]^-^ | 429.31 | 7.86 | 385, 249, 205, 279, 161 | Monoaryl |
| Kaempferol 3-glucuronide | C_21_H_18_O_12_ | [M-H]^-^ | 461.11 | 7.89 | 285 | Flavonoid |
| Quercetin 3-O-rhamnoside | C_21_H_20_O_11_ | [M-H]^-^ | 447.22 | 7.90 | 301, 300 | Flavonoid |
| Diarylheptanoid-hexose | C_25_H_30_O_9_ | [M-H]^-^ | 473.21 | 8.27 | 293 | Diaryl |
| Quercetin-3-O-galloylhexoside | C_28_H_24_O_16_ | [M-H]^-^ | 625.34 | 8.32 | 607, 463, 415, 397, 301 | Flavonoid |
| Quercetin 3-hydroxybenzoylgalactoside | C_28_H_24_O_14_ | [M-H]^-^ | 583.25 | 8.34 | 463, 301 | Flavonoid |
| Kaempferol O-pentoside | C_20_H_18_O_10_ | [M-H]^-^ | 417.22 | 8.45 | 327, 285, 255 | Flavonoid |
| Phenethyl rutinoside | C_20_H_30_O_10_ | [M-H]^-^ | 429.31 | 8.58 | 385, 285, 249, 205 | Monoaryl |
| Kaempferol-3-O-rhamnoside | C_21_H_20_O_10_ | [M-H]^-^ | 431.17 | 8.58 | 327, 285, 255 | Flavonoid |
| 1,7-bis-(3,4-dihydroxyphenyl)hept-4-ene-3- one | C_19_H_20_O_5_ | [M-H]^-^ | 327.18 | 8.74 | 283, 271, 253, 241, 211 | Diaryl |
| Myricetin | C_15_H_10_O_8_ | [M-H]^-^ | 317.08 | 8.82 | 287, 271 | Flavonoid |
| Quercetin 3-(2''-p-coumarylglucoside) | C_30_H_26_O_14_ | [M-H]^-^ | 609.14 | 9.02 | 463, 301 | Flavonoid |
| Quercitin | C_15_H_10_O_7_ | [M-H]^-^ | 301.05 | 10.20 | 227, 209, 183, 165 | Flavonoid |
| Apigenin | C_15_H_10_O_5_ | [M-H]^-^ | 269.11 | 11.26 | 225, 201, 183 | Flavonoid |
| Trihydroxymethoxyflavone | C_16_H_12_O_6_ | [M-H]^-^ | 299.12 | 11.41 | 284, 255 | Flavonoid |
| dihydroxy-dimethoxyflavone | C_17_H_14_O_6_ | [M-H]^-^ | 313.14 | 13.03 | 298, 269, 227 | Flavonoid |
| dihydroxy-dimethoxyflavone | C_17_H_14_O_6_ | [M-H]^-^ | 313.18 | 14.51 | 298, 269, 227 | Flavonoid |
| dihydroxy-dimethoxyflavone | C_17_H_14_O_6_ | [M-H]^-^ | 313.14 | 14.99 | 298, 269, 227 | Flavonoid |

**Table S2**: Mean values of measured traits for silver birch genotypes ‘O154’ and ‘36’ growing in single genotype plots. *Phenolic compounds quantified using LC-MS by calculating cumulative peak areas in reference to chrysin, which was added to all samples at a concentration of 0.01 mg/mL and was assigned a peak area of 1.

| Measurement | O154 mean value ± SD | 36 mean value ± SD |
| --- | --- | --- |
| DBH (cm) | 13.81 ± 2.52 | 11.63 ± 2.93 |
| SLA (g/cm^2^) | 15.13 ± 1.75 | 15.16 ± 1.20 |
| Herbivory | 1.66 ± 0.95 | 4.12 ± 1.57 |
| Total phenolics* | 7.90 ± 0.57 | 8.86 ± 0.41 |
| Flavonoids* | 4.90 ± 0.32 | 5.92 ± 0.28 |
| Quercetins* | 2.28 ± 0.26 | 2.72 ± 0.24 |
| Myricetins* | 1.73 ± 0.13 | 2.17 ± 0.15 |
| Kaempferols* | 0.31 ± 0.03 | 0.38 ± 0.06 |
| Condensed tannins* | 0.78 ± 0.07 | 0.86 ± 0.09 |
| Hydroxycinnamates* | 1.97 ± 0.21 | 1.78 ± 0.10 |


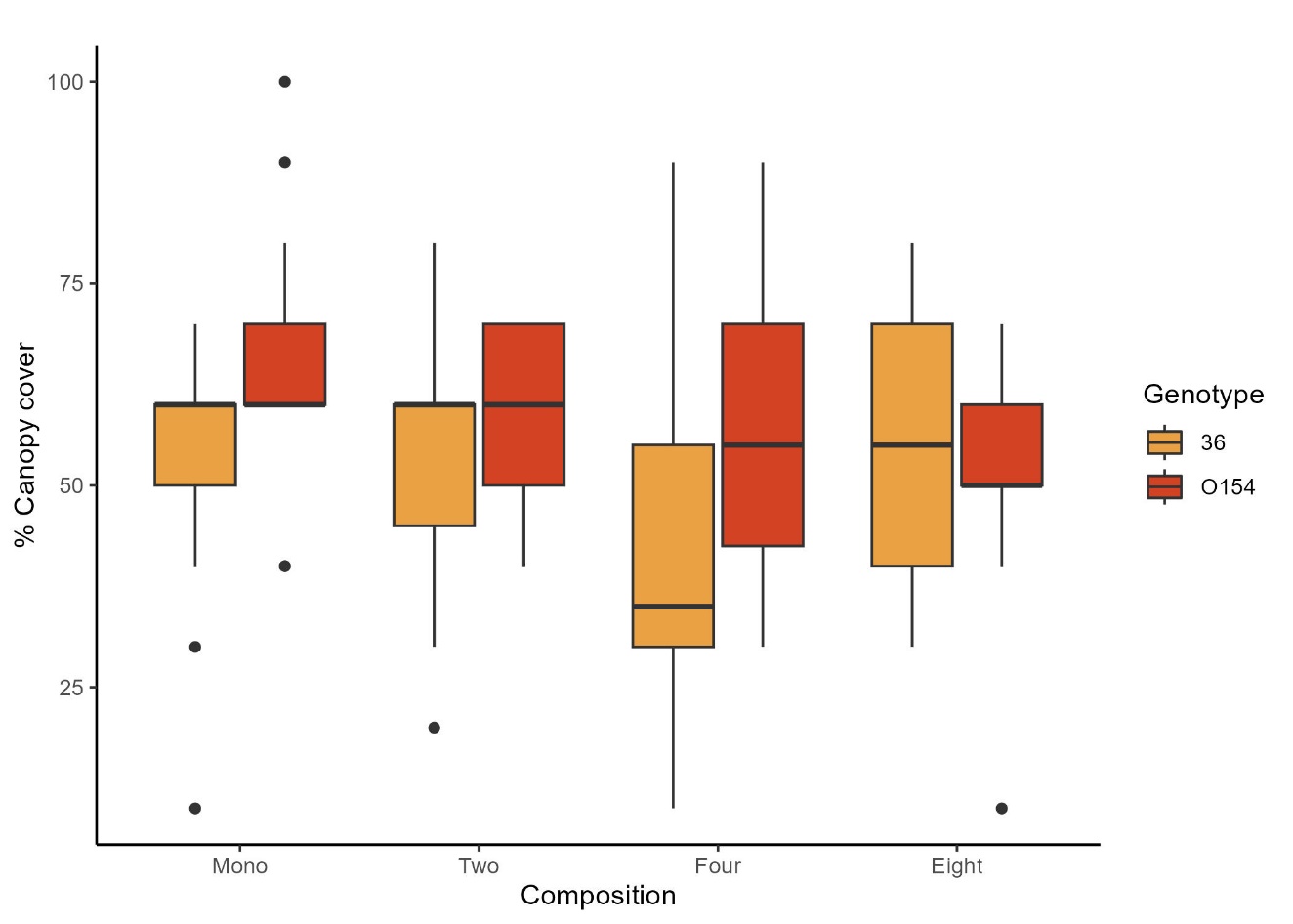


**Figure S2:** Canopy cover around focal trees of two birch genotypes growing in monocultures and, 2-, 4-, or 8-genotype plots

**
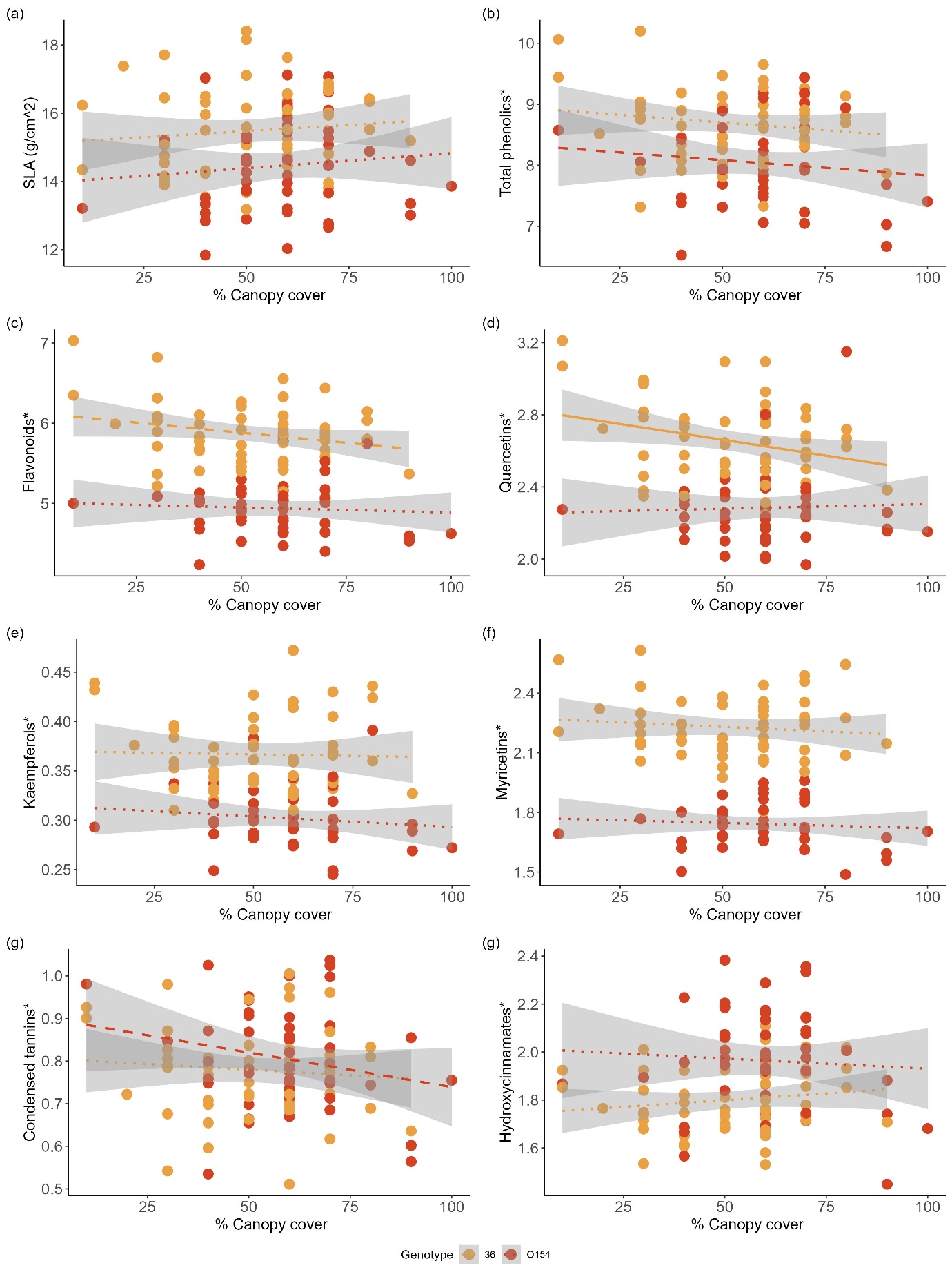
**

**Figure S3**: Regression plots showing the influence of % canopy cover on birch leaf traits. Dotted lines indicate non-significant relationships, dashed lines indicate marginally significant relationships, and solid lines show significant relationships.
